# Supplementary material for: A feedforward mechanism for human-like contour integration
Source: PLoS Comput Biol. 2025 Aug 18;21(8):e1013391. doi: 10.1371/journal.pcbi.1013391 (PMC12370188; doi:10.1371/journal.pcbi.1013391)
Supplement: Attachment — Submitted filename: rebuttal_letter_response_to_reviewers.pdf [file pcbi.1013391.s015.pdf]

HARVARD UNIVERSITY
DEPARTMENT OF PSYCHOLOGY

WILLIAM JAMES HALL
33 KIRKLAND STREET

Fenil R. Doshi
fenil_doshi@fas.harvard.edu

Dear Editor,
PLOS Computational Biology,
March 17, 2025

Dear Editor,

Thank you for reviewing our manuscript, "***A feedforward mechanism for human-like contour integration***" (PCOMPBIOL-D-24-01619). We have responded to all three reviewers' comments and have included our revised manuscript.

In particular, we have

- Conducted new analyses to evaluate the generalization of fine-tuned models across different contour curvatures (Supplementary Fig. 11)
- Verified the key results with the complete participant set (Supplementary Fig. 12)
- Made changes in Fig. 3 to show the receptive field progression in the models more clearly
- Expanded the results section "*Progressive receptive field growth with layer depth underlies the model's ability to support contour integration*" to clarify the value of pinholenet experiments
- Refined our discussion of fine-tuning throughout the revised manuscript to emphasize its role in the emergence of human-like contour integration.
- Expanded our discussion to better contextualize our findings within the broader literature, particularly in relation to perceptual grouping, uncrowding, and the role of feedforward mechanisms.
- Addressed the relationship between human and machine vision, clarifying what can be directly inferred from our findings and what remains speculative
- Refined key statements throughout the manuscript (including the Abstract) to ensure clarity and accuracy in presenting our claims.
- Clarified key methodological details, including stimulus generation, model evaluation, human experiment design, and statistical analyses
- Made additional minor revisions in response to several reviewer comments.

Please find our point-by-point response to the reviewer's comments, with changes to the manuscript marked in blue. Thank you for your consideration. Please let us know if there is any further information you need.

Sincerely,
Fenil R. Doshi, Talia Konkle and George A. Alvarez

Contact Information:
Fenil R. Doshi fenil_doshi@fas.harvard.edu +1 (224) 499-4442

Talia Konkle	talia_konkle@harvard.edu	+1 (617) 308-6291
George A. Alvarez	alvarez@wjh.harvard.edu	

POINT-BY-POINT RESPONSE TO EDITOR AND REVIEWERS
*** Reviewer comments in black and response in red**

Reviewer #1:

The authors explore the possibility that path detection and contour integration can be achieved through purely feedforward processes by finetuning an ImageNet trained AlexNet to do the path detection task. Results suggest that paths can be accurately detected by finetuning networks for the task but that networks trained purely for object recognition do not accurately detect these paths. The authors also report evidence that DCNNs' performance on the path detection task (as a function of turn angle between consecutive Gabors) most closely correlates with humans when networks are finetuned with a turn angle of 20 degrees. Better-performing neural networks like
ResNet and ViT do not get path detection for free from their more sophisticated architecture.

I liked this paper quite a bit and think it has great potential to be widely cited by vision researchers. I agree with the authors' approach of using neural networks not as models
of visual perception but as tools for testing what is computable with the kinds of architectures by which networks are built. The authors offer a compelling existence proof that feedforward mechanisms are on their own sufficient for humanlike contour integration. I do have a few suggestions that I think would strengthen the article and that I would like to see the authors address before I recommend it for acceptance.

Thank you for your thoughtful and encouraging feedback on our work. We are delighted that you found the study compelling and see its potential impact within the vision science community.

First, I was curious if the authors played with minimally misaligning Gabors to test whether small adjustments to spatial position in the local elements have the same large
effects on path detection for neural networks as they do for humans. The authors mentioned that they minimally changed displays by changing the orientation of elements in
the contour path, but it might be easier to detect that two elements are compatible or incompatible in terms of their orientation than in terms of their spatial alignment. If two
contours have compatible orientation but their positions are slightly jittered so that they do not form a path, will networks fail to see a path as humans do?

We didn't develop any display conditions that qualitatively disrupt human perception of the contour using controlled variation in spatial position. Our displays did have some spatial position jitter, following the same procedures as Field and colleagues.

Noteably, empirically we observed substantial display-by-display variability in human perception, even *within* a contour curvature condition, with some displays being easier and others harder to detect the contour, as seen in Figure 4D. Some of this display level variability might happen due to the spatial jitter or other random aspects of the display generating process. Whatever the cause, models were evaluated for their emergent match over display-by-display level variation, so must account for differences in perception beyond the curvature condition across the full span of detectability.

We have also clarified our jitter parameters in the manuscript (in the "*Stimuli Generation*" Subsection of Methods; line 820-824; line 838-849), and updated Supplementary Figure 1 for clarity: When spatially positioning each Gabor into a grid cell, path elements were not centered in the grid, but were instead placed at the midpoint of the two anchor points, leading to spatial jitter within each cell. For background elements, spatial jitter was included, displacing elements by up to 9 pixels in both the x and y directions. Note that the spatial position and orientation of each background gabor element was identical between the matched 'contour-present' and 'contour-absent' images."

I would also be interested to see a comparison in the effect of Gabor density on human and DCNNs' sensitivity to the contour path. I know that the authors' control stimuli equated density, but I am curious about the role density plays in displays with a perceptible path. Does path detection fall off more or less quickly as a function of inter-element distance in neural networks compared with humans?

This is great question—and is made difficult by two facts: (1) isolating density from element count and number is non-trivial, and (2) human acuity falls off with eccentricity. For example, if you want to decrease density without changing the number and size of elements on the display, they you need to present the less-dense stimulus at a bigger visual angle--this is in fact exactly the choice that Field et al., did when the explored inter-element spacing effects. They found an interaction, with reduced contour detection at the curviest paths, with accuracy worsening as the display became less dense. However, they note this effect is confounded with peripheral acuity and they highlight that it is difficult to de-confound one factor without confounding others (e.g. you could keep the same display size, but then you'd have to either decrease the number of elements on the path, or make the elements smaller). Because of these complexities, we haven't pursued this signature here; maybe models that explicitly have a fovea-periphery acuity gradient are better testbeds for understanding density signatures. We now list density among the properties that should be studied, under the "*Limitations of the approach*" Discussion section (line 664).

Minor:
I was a little concerned by the authors' criteria for excluding data from the human experiment on the basis of its dissimilarity to other participants. Do any of the important findings from the paper change if the authors include these excluded participants?

Thank you for raising this concern. To examine the impact of our exclusion criteria, we conducted a re-analysis including all 108 participants. The results show that the overall conclusions hold, which we now state in the manuscript (line 1032-1033), and a new Supplementary Figure 12.

- **The human noise ceiling is at 0.774 (±0.011). for all subjects vs. 0.785 (±0.009) for selected subjects.**
- **The broadly-tuned model (average pool layer) still shows a lower correlation with human data (all subjects r=0.186; vs selected subjects: 0.197).**
- **The controlled models that are narrowly-tuned, demonstrate higher alignment with human behavior, peaking when trained with 20° contours approaching the noise ceiling (all subjects r=0.766; vs selected subjects: 0.768).**

To illustrate this, we have included the figure below, which shows the correlations between human performance and the narrowly-tuned models (fine-tuned from the avgpool layer) across different

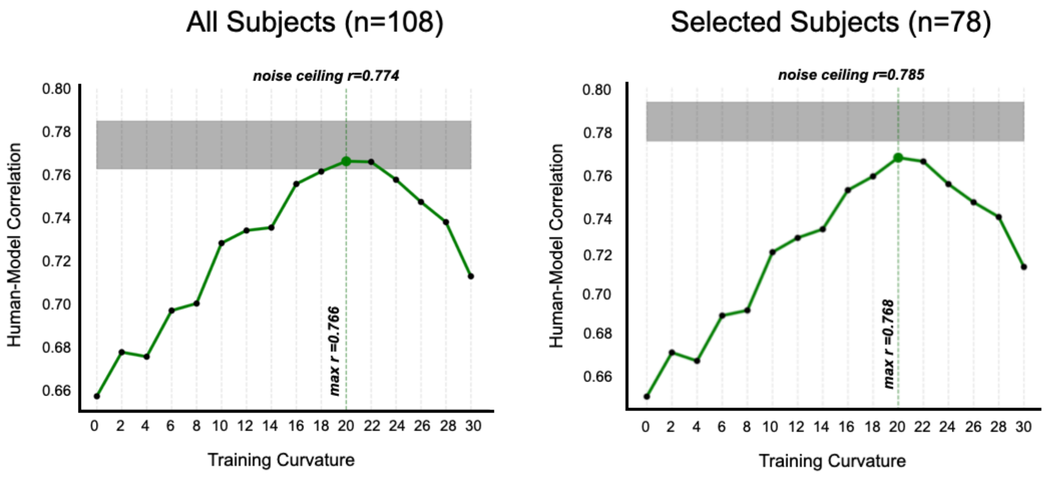

training curvatures. The left panel represents correlations using the full participant set (n=108), while the right panel shows correlations for the selected subset (n=78). Despite minor differences in absolute values, the overall pattern remains consistent—human-model correspondence peaks when models are trained with 20° curvatures. The noise ceiling (shaded region) is also nearly identical across the two subject groups (though slightly higher within the selected-subjects group), reinforcing the robustness of our findings and demonstrating that the exclusion criteria have minimal impact on the key conclusions.

I am unclear on how the authors computed the 95% confidence intervals in Figure 2. Perhaps I missed this in the Methods. Could they explain this more?

Thank you for raising this question. The 95% confidence intervals (CIs) in Figure 2 were computed using predictions from a held-out test set of 600 images. First, the model classified each test image as either containing a contour or not. Each prediction was then evaluated for correctness, generating a binary list (1 for correct, 0 for incorrect). The mean accuracy and the standard deviation were calculated across all 600 test images using this correctness list. Finally, the 95% CI was computed using the following standard equation:

$$CI = 1.96 \text{ x } {std_{dev}}/{\sqrt{N}}$$

where N=600 is the number of images in the held-out test set

This method ensured that the CIs reliably captured variability in prediction accuracy, offering robust performance estimates for each layer across the held-out test set. These details are now clarified in a new "*Confidence Interval for Test Stimuli*" subsection of Methods in the revised manuscript (line 906-914).

Line 193: Figure 2B should be Figure 2A

FIXED!

Line 225: "The model relies on fine-grained collinearity between contour elements to isolate the contour from the background." Aren't there many elements that are not collinear that still organize together into a path? Unless I am misunderstanding, reliance on collinearity seems to strict for what humans and networks are both doing here.

We see how our use of the term "collinearity" may be misleading if it is interpreted strictly as 'in a straight line', rather than, 'along a smooth path'. We have revised the text to replace "fine-grained collinearity" with "fine-grained orientation information (line 270)." We also replaced this term in line 615 with "local-alignment".

Line 293: There is a typo here. "…while still able to succeed at object recognition" □ "but still being able to succeed at object recognition".

FIXED!

Line 362: "were able to accurately detect relative straight contours" □ "were able to accurately detect relatively straight contours"

FIXED!

Line 427: "…that we are not directly fit the model" should be "that we did not directly fit the model" or "that we are not directly fitting the model"

FIXED!

Line 471: "In an auxiliary analyses" should be "In auxiliary analyses" or "In an auxiliary analysis"

FIXED!

Reviewer #2:

This study examines the applicability of feedforward network architectures to a well-known psychophysical paradigm (informally known as "snake stimuli") that
was popular in the 1990s for studying contour integration in human vision. The main finding is that these architectures can perform the task associated with this paradigm.
Furthermore, under some conditions, they can mimic some aspects of human behavior.
When I first read the abstract of this paper, I was excited: it suggested to me that this paper challenged a bold proposal put forward by two articles, one in PLoS Comp Biol
(this journal) and one in Vision Research, by Doerig et al:

- Doerig A, Schmittwilken L, Sayim B, Manassi M, Herzog MH (2020) Capsule networks as recurrent models of grouping and segmentation. PLoS Comput Biol 16(7):
e1008017).
- A. Doerig, A. Bornet, O.H. Choung, M.H. Herzog, Crowding reveals fundamental differences in local vs. global processing in humans and machines, Vision Research, Volume
167, 2020, Pages 39-45.

The second article makes a claim that is directly relevant to the counter-claim made in the present manuscript: "Our results provide evidence that ffCNNs cannot produce
human-like global shape computations for principled architectural reasons." (from the Abstract of Doerig et al. Vision Research). The "ff" in ffCNNs stands for feedforward.
Doerig et al proposed that ``uncrowding," a counterintuitive phenomenon in human perceptual grouping, cannot be explained by feedforward architectures. Because the
perceptual grouping processes that support uncrowding also involve contour integration and segmentation, and because the claims by Doerig et al are closely related to those
made in the present ms, this prior work seems directly relevant to the submission under review.
Disappointingly, when I accepted to review it on the back of that initial excitement, I realized that this study focuses instead on a different phenomenon in perceptual grouping,
and one that has lost much of its interest for a good part of the past 20 years. It was popular before 2000, but only sporadically seen in the perceptual literature these days.
There is no mention of prior work on uncrowding, a much more interesting phenomenon that continues to challenge current ideas on grouping/segmentation and their relation
to vision models in machine learning.

Thank you for highlighting this relevant work by Doerig et al. (2020). We have revised the manuscript to include citations of these papers in the introduction (line 108, 112), and a discussion of these results and the relationship to ours in the general discussion (line 668-675).

More to the point with specific reference to the central claim of this manuscript, nobody in 2024 with minimal familiarity with the work that has been carried out over the past
two decades would think it reasonable to advance the notion that *no* DNN can solve snake stimuli, or more generally perform good continuation, or more specifically reproduce a human behavioral trace consisting of 5 data points (Figure 5F). When the authors stage this position as the accepted claim they set out to challenge, they are setting up a straw-man argument: they refer to a paper published by Field et al. in 1993 (lines 82-84), i.e. 30 years ago. Nobody today would think it reasonable to make such a claim (and even what Field et al. meant is not exactly what the authors of this paper claim they meant, but that is not the sticking point here). Technically speaking, the paper is perfectly adequate: it goes through standard alignment procedures for testing compliance with human behavior. There is no material that strikes this reader as particularly innovative, but nothing to complain about either: everything is executed well and in line with current standards. The problem is that, as I hinted at above, the primary finding is completely unsurprising: of course feedforward architectures can do this, of course they can do it more or less well depending on how much good continuation bias is injected into the learning diet, whether directly (fine-tuning) or indirectly (pre-training), of course having increasingly larger RFs is conducive to solving a problem that requires long-range integration over space. What is surprising about these results? Perhaps that, under some interpretative extrapolation of what others may have thought (or not) three decades ago, they now prove them wrong in 2024? I fail to understand the relevance of this result today.

Thank you for your thoughtful feedback. We will first address an apparent confusion about our model-human comparison procedure, and then address comments regarding whether anyone would find our work surprising.

First, you imply that we are simply "reproduce[ing] a human behavioral trace consisting of 5 data points". However, this is incorrect: our critical measure is the model's emergent correlation to human data from 1,000 points, at a trial-by-trial granularity (clarified in the revised manuscript; line 474-475). We can see how Figure 5 could be misleading (it is included as it aggregates the trials by degree of contour curvature, following conventions of the original Field et al paper).

Second, we want to also clarify that the model-human performance correlations are *emergent*--that is, we never trained the model on any human data ever. Across the sweep of controlled variation over dozens of models, some models showed more human-like signatures (e.g. those trained to detect 20-degree contours), while others show less human-like signatures (e.g. those trained to detect purely straight contours, or those trained to detect contours with a wide range of curvatures from 0 to 75 degress). In other words, we use the set of matched models that vary in controlled ways to explore what priors lead to the most emergent human-like contour detection signatures), leveraging patterns of success and failure for inferential purchase. We never directly train a model to reproduce the human data. It could have been the case that none of our model variations were successful in reproducing the human trial-by-trial patterns of results—this possibility is in fact part of what allows there to be a discovery here (rather than just showing models can capture data they are trained on).

We have re-worded several sentences throughout the "*Fine-tuning to detect low curvatures gives strongest alignment with human behavioral data"* section of Results, to hopefully add more clarity about these methods. Reviewer 3 had a similar comment.

To your next critique, you say: "nobody with minimal familiarity with this work would thinking it reasonable to assume that [feed forward CNNs] can't solve snake stimuli." Honestly, we ourselves did, and would like to think we have at least minimal familiarity with the work ;). Specifically, we thought that Alexnets would be architecturally incapable of detecting these contours, much like the claim you highlighted in Doerig's work that "their results provide evidence that ffCNNs cannot produce human-like global shape computations for principled architectural reasons." And, at the outset of this project, we thought that this simple task and likely failure mode would then provide a good starting point for exploring what other mechanisms are needed to add to bring about this capacity (like lateral connectivity and recurrence in early layers). So, in truth we had other plans for the directions of this project, but the surprising results (to us) lead us down this path of instead having to figure out how these purely feed-forward networks were in fact succeeding! Which lead us to discover that it was later stages that were accomplishing contour integration, which we then verified with the pinhole networks. So, we humbly disagree that no one would be surprised by these findings—as we were! (And, neither of the other two reviewers suggested this work was a strawman, or unsurprising).

To be absolutely clear: I think these results should be published, but (in my opinion) not in PLoS CB, or at least not in their current form (see further below for suggestions).
The study is technically well-conducted, and it explores a psychophysical phenomenon that has not been addressed before in this way, but if we ask precisely why it hasn't been studied in this way by others before, the answer in my mind is clear: in 2024, it is simply not interesting to choose this particular phenomenon to exercise the power/capability of DNNs. That is why previous investigators interested in this question have set out to demonstrate the power and limits of DNNs with reference to much more challenging problems, such as uncrowding (see material cited above).

In conclusion, and in an effort to provide constructive criticism and offer suggestions for improvement, if the authors are able to revise this study and demonstrate that feedforward architectures can capture human behavior for a challenging problem like uncrowding, then I would be happy to support (in principle and following evaluation that the claim is justified) publication in PLoS CB. But as the manuscript stands now,

i.e. limited to the particular phenomenon of contour integration in snake stimuli, I don't see how this study can make the threshold for a journal like PLoS CB, at least insofar as my (high) opinion of this journal is concerned.

We hope that the clarifications discussed above about how we were testing the capacities of these models, e.g. the fact that we never train on human data and we look for emergent fit of 1000 trial-by-trial perceptual decisions, helps to alleviate some of your concerns about what we were testing and thus what are claims are. We also hope that the quality of the care taken in the process, including sweeps of models only some of which showed emergent correlation with the human data, and the introduction of pinhole nets to really nail down how the contour detection capacity was arising (while also dissociating it from object recognition capacity; see our responses to Reviewer 3) makes this work a worthy and quality scientific contribution.

Reviewer #3:

The authors assess the extent to which a standard CNN captures characteristics of human contour-integration, and what modifications are required to coax the net into showing more human-like behavior. The key results are that standard CNNs do not show emergent human-like contour integration and that fine-tuning on the task is required. The authors suggest that their data show that in order to reach high accuracy on contour integration, the typical progression from small to larger receptive fields is important during fine-tuning. Moreover, they suggest that in order to recapitulate the characteristics of human contour-integration in response to different types of contours (i.e., contours with different beta parameters), an inductive bias towards relatively small beta parameters is required during fine tuning. Overall, I enjoyed reading the manuscript. It's very well written, mostly clear, and refreshingly cautious in its conclusions. I also believe that the authors' approach is useful and worth being published. However, I have a few comments/concerns, which the authors will hopefully find useful.

Thank you for your thoughtful and constructive feedback on our manuscript. We are glad to hear that you found the study well-written and clear!

General/major points:
- I have some reservations about the extent to which the authors really show that fine-tuned models show human-like performance (see below). However, for the sake of the argument, let's assume that their fine-tuned models do show human-like performance. In my view, there is one important point that gets lost throughout the interpretation and discussion: the inductive biases highlighted in this paper don't lead to emergent human-like performance on contour detection. Rather, these inductive biases have to be used during fine-tuning in order to give rise to human-like performance. I'm a bit at a loss about what that result tells us about human vision, machine vision, or the relationship between the two. The authors show that in order to achieve human-like performance, they have to use certain inductive biases during fine-tuning. But the human visual system is (presumably) never fine-tuned in the same way. So, I'm unsure what we are to make of the inductive biases that are required during a type of training to which the human visual system is never subjected. Maybe the authors could clarify their views on this issue? Isn't it possible that the inductive biases identified here are only important for the specific type of fine-tuning used in this paper, but not for the kind of learning that happens in the human visual system?

Thank you for raising these important questions. Our study has the strongest grounds to make claims about machine vision, that is: certain architectural and training conditions, such as progressive receptive field growth and sensitivity to smaller beta parameters, enable feedforward networks to achieve human-like contour integration performance. However, as you highlighted, it would be a mistake and quite confusing to claim that the human visual system learns to do contour detection in the same way as the model. Instead, the next sound inference we can make is that, since a model with only a feed-forward hierarchy can *in principle* succeed at contour detection, if they have sufficient progressive receptive field structure. Thus, it's possible that later stages of human visual processing (e.g. IT!) play a key role in contour integration, which departs from standard focus on early area interactions (e.g. V1, V2).

Next, the fact that some models can show emergent human-like signatures with some fine-tuning task biases (i.e. 20 degree curves) but not others (i.e. 0 degree straight lines), permits the next safe inference: maybe the human visual system has a specific mechanisms to amplifies local contour alignment around 20 degrees. But, we do not want to imply that task-specific fine tuning is the inducing mechanism; and indeed this claim about a 20-degree specialization is only consistent with, but certainly not required by, these results. Instead, this speculation provides a rather targeted mechanism that one could explore behaviorally and with new model development (e.g. perhaps a model armed with such a local curvature amplification mechanism might not need task-based fine-tuning anymore!).

We have modified our General Discussion to clarify our view. To put it succinctly here: what we learned about *models* is that training on object recognition (ImageNet classification) does not

automatically build human-like contour integration capabilities, but that additional biases are needed that accomplish something like "amplifying gradual ~20degree curves" (line 597-605; also see "*Towards Emergent Human-like Behavior*" subsection of Discussion for additional details). What we learned about *human vision* is that the late stages of the visual hierarchy, e.g., area IT, may be more involved in contour perception than assumed by research and models that focus on early (V1, V2) stages of processing. (line 647-651).

- I think the abstract and parts of the discussion are not ideal reflections of the work. For instance, in my view, a sentence such as 'We identify two key inductive biases that give rise to human-like contour integration in purely feedforward CNNs' is misleading. It's not the inductive biases that give rise to human-like contour integration. It's the fine-tuning on the task that's required first and foremost! I think the abstract (but also some parts of the discussion) would provide a better summary of the work if the authors focused more on the finding that current CNNs do not show emergent human-like behaviour, and that for fine-tuning, certain inductive biases are helpful.

We very much appreciate your point here—and agree that the fine-tuning part needs to be more clearly emphasized, along with its implications for what we can claim. We have now edited the manuscript in several places, including the Abstract (line 34-49), Introduction (line 130-132; line 140-141) and Discussion (line 597-602). For example, we no longer summarize this work as identifying 'two key inductive biases' (though we highlight the importance of these key factors in the text), and we now highlight that (biased) fine-tuning is needed for human like behavior. We have looked through our use of the word 'inductive bias' throughout the manuscript to be more careful, and call-out the fine-tuning aspects more directly. We think this is a better reflection of the work's contributions now—thank you for how clearly you were able to point this out.

- Regarding PinholeNets: I'm not sure how surprising it is that these networks aren't able to detect contours. They are set up not to be able to process the relevant information from the input due to the restriction in receptive field size. I hope I'm not being unfair but I'm not sure how interesting this finding really is. Could the authors explain in more detail in what sense the finding is not trivial?

In the revised manuscript, we've now clarified the logic of these analyses, including modifying Figure 3 to show the receptive field progression across all the models being compared, highlighting that all models including PinholeNets have full-field receptive fields in the final fully-connected layers (line 303-308). All changes are reflected the "*Progressive receptive field growth with layer depth underlies the model's ability to support contour integration*" section of the revised Manuscript (line: 303-305; 329-347; 359-361).

To elaborate here, we think the results are non-trivial, in the following ways: When fine-tuning from the Conv5 backbone, reading out directly from the feature backbone, the pinhole models all performed relatively poorly. This result indicates it's not easy to read out a contour from a relatively high spatial resolution (small RF) encoding of the image. Instead, the relatively low spatial resolution (large RF) outputs of the AlexNet backbone support better contour detection, suggesting that the more the learned features have to pool over space, the better.

Second, the complete pinhole model does not actually lack global information: the later stages do have access to full-field information— like AlexNet they actually have a 6x6 AdaptiveAvgPool and two fully connected layers after their convolutional backbone, which aggregate information from the entire image. The fully-connected layers have full-image receptive fields that certainly encompass the entire contour. We also report results after fine-tuning the model from this later stage; only the two largest PinholeNets show successful contour detection abilities (whose final conv layer RFs span more than one element of the contour in the displays). These results indicate that it is not sufficient to have full-field receptive fields in order to be able to perform the contour detection task well; instead, this

result indicates that larger receptive field sizes enroute to those fully-connected layers is important. We hope the revised section now clarifies the contributions of the PinholeNet model experiments.

- Related to my previous point, I'm wondering whether the authors can really say that a progressive increase in receptive field size is required. Maybe all that's required is a receptive field size large enough to cover the spatial extent of the contour. For instance, I'm wondering what would happen to contour detection performance if the receptive field for the PinholeNet would be 195x195. I think the existing data already might hint at a possible result: contour detection performance increases steadily from the PinholeNet with the smallest to the largest receptive field (as seen in Fig. 3C, left panel). My expectation is that, as receptive field size increases further, performance would improve as well. If so, I think that's a clear indication that what matters is not a progressive increase in receptive field size but a size large enough to allow the model to process the relevant characteristic of the input.

Following from the previous answer, we now directly show the receptive field progression in the models tested in Fig. 3A of the manuscript, so it's clearer what we mean by this term. In the interest of avoiding too much nuance we didn't try to change the phrase "progressively increasing" but instead have attempted to clarify the fact that there are fully-connected layers at the end of the pinhole nets that could in principle pick up on contours. (line: 303-308; 337-347; line 359-361).

Second, we also now describe the pattern of results more carefully line 337-347: the two smallest pinhole nets can only ever encompass one gabor before the fully-connected combination, whereas the next two bigger pinhole nets span a little more than 1 gabor, and show better contour detection accuracy. Alexnet shows higher accuracy still. (Testing a PinholeNet with a 195×195 receptive field in its convolutional layers would be interesting, but it is computationally infeasible due to the high parameter count--akin to doing fully-connected layers at every stage over the full image). So, more generally we hope that this new section more clearly highlights how having less spatial resolution but features that pool from a larger receptive field (like the standard alexnet) is actually more effective for detecting contours than reading out from a high spatial resolution buffer.

- I'm slightly confused about the beta values of the different stimulus levels used in the human observers and the models. I have tried to gather the information together and believe the authors used 6 stimulus levels to assess models (beta of 0 to 75 deg in steps of 15 deg). For human observers, it seems only 5 stimulus levels were used (15 to 75 deg in steps of 15 deg). Is that correct? First, I think this needs to be made much clearer, it really confused me and I unnecessarily wasted time trying to gather the relevant information.

Apologies for the confusion in the methods; you correctly figured this out: In the human experiments we followed prior work by Field et al and tested displays generated from five beta values (15°, 30°, 45°, 60°, and 75); but, while separately evaluating the models contour detection capabilities (i.e. Fig. 2 and Fig. 3), we used distinct displays generated from six beta values – 0°, 15°, 30°, 45°, 60°, and 75°, averaging across all of these displays to estimate it's overall contour detection performance. We have revised the "*Behavioral Experiment*" section of Method to explicitly describe these details (line 984-990).

More importantly, I think the choice of stimulus levels for the humans might be problematic. Humans are close to perfect with contours that have a beta value of 0. I would be surprised if the model that was trained on contours with a beta value of 20 deg, would match this human performance. However, in order to make strong claims about the training with 20 deg contours, it would be critical to show that the model matches human performance not only on the chosen stimulus levels but also at 0 deg. This is particularly important because it's not really surprising that a model trained on

contours with beta of 20 deg performs best in response to contours with a beta of 15 deg, if the only other tested contours have beta values that are further off.

To illustrate my point, assume I have two stimulus levels, one with a beta of 15 and one with a beta of 75. Would it be surprising that a model trained on contours with a beta of 20 performs better with the 15 deg contours than the 75 deg contours. Of course not! But if training with 20 deg beta values leads to better performance on 0 deg stimuli compared to 15 deg stimuli (as I expected would be the case for humans), that would be very surprising in my view. At the very least, I would like to see model performance in response to 0 deg stimuli in the model trained with 20 deg stimuli (in the plot in Fig. 5F).

Prepare to be surprised! We understand your question here to be about how well the 20°-fine-tuned model generalizes to the 0° beta displays. You're highlighting that the model that was fine-tuned on 20° contours might have a natural fall-off of accuracy around that point, with ok accuracy at 15 degree contour displays (-5 degrees off), and even worse at 0-degree contour displays (-20 degrees off). And your intuition (which we agree with, though we didn't measure) is that humans will be quite good at 0-degree displays.

To explore this, per your suggestion, we tested the 20°-fine-tuned model on held-out test set displays from 0-75 degrees, by 1 degree increments (See Figure below). We find that the 20°-fine-tuned model has asymmetric generalization! That is, it is quite good at displays from 0°-20° contours, and then falls off after that.

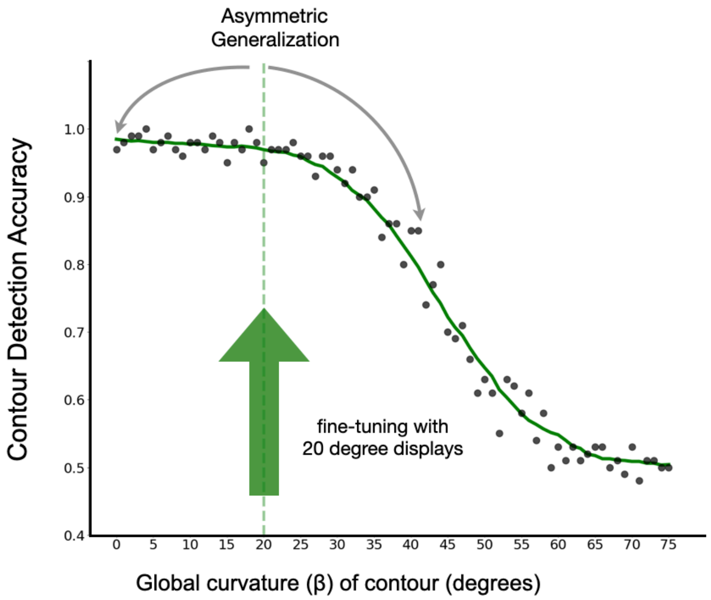

We have not added the 0° condition to figure 5F because this figure is about human-model comparison and we didn't test humans at 0°. Instead, we now include a new supplementary figure (Supplementary Fig. 11), and highlight these results in the manuscript (in "*Fine-tuning to detect low curvatures gives strongest alignment with human behavioral data*" subsection of Results; line 505-508).

- Related to my previous point, it seems that this sentence makes little sense: "For example, the model trained on 20 deg curvature might show poor contour detection accuracy at 0 deg, 10 deg, 15 deg, and 25 deg etc." We are never shown how the model performs at 0 deg, 10 deg or 25 deg.

In the revised manuscript, we have revised this section ("*Fine-tuning to detect low curvatures gives strongest alignment with human behavioral data*" section) and now show performance over the whole beta range from 0 to 75 in one degree increments in the new Supplementary Figure 11 and highlight these results in the Revised manuscript line 505-508).

- I don't understand how this sentence is linked to the actual analyses: "…our analyses revealed that fine-tuning with an inductive bias towards gradual curvature was required to see more human-like contour detection decisions." I think whatever the idea here is requires more unpacking (at the least, you need to refer the reader to the specific analysis that supports this statement).

Thanks for highlighting this; we have revised this section ("*Fine-tuning to detect low curvatures gives strongest alignment with human behavioral data*" section; line 464-475; 496-508). Specifically, this statement follows from the key result that models fine-tuned on contours with relatively gradual curvatures (20°-beta) showed the highest emergent display-by-display level correspondence with human judgements; while, critically, models fine-tuned with contours <20° or >20° did *not* show as clear an emergent match. We have clarified this following that sentence, line 597-602.

- I'm not sure I understand what the authors have in mind with these sentences:
o 1. "Thus, these results provide a clear direction for future research — to discover new inductive biases that can be built into DNN models so that human-like contour detection emerges without any fine-tuning to the contour task." Why would that be useful? Why would we want to build these inductive biases into DNNs? In my view, the emergence of human-like contour detection without fine-tuning is not an objective per se. More importantly, the authors haven't shown that building these inductive biases into DNNs doesn't require fine-tuning. They have only shown that fine-tuning with specific inductive biases leads to more human-like behaviour on the task for which the model has been fine-tuned.

We see the confusion we caused; it's also related to your earlier point that the importance of (biased) fine-tuning needed to be emphasized to situate the results better. In the revised manuscript, we have clarified this in the General Discussions (line 597-605) and highlighted the importance of this point in the "*Towards Emergent Human-like Behavior*" section of Discussions.

To elaborate here, one striking result would have been if a model trained only on natural images for object classification automatically showed emergent human-like contour detection signatures without any additional fine-tuning. We didn't find that. Instead, we found that pretraining on natural image statistics provided some contour detection capacity (moderate transfer, orange dots in Fig. 2), but that the models showed a rather striking emergent match to human signatures with (biased) fine-tuning, specifically at 20°.

So, to us this is useful, e.g. it suggests we could try building a local feature-association mechanism focused/tuned to 20° curvature into the architecture itself, and then perhaps natural image statistics and higher-order classification tasks will be sufficient to show emergent human contour detection matches without any fine-tuning.

o 2. "For example, perhaps more human-like contour detection will naturally emerge in in models trained on even more general visual objective (e.g., self-supervised learning or predictive coding over more 3-dimensional object views.)." Maybe. But I'm not sure how this relates to the current paper. Which part of your data supports this idea?

"Maybe" is right; here we are simply speculating about what visual data and task objectives might lead to fully emergent human-contour signatures (without requiring fine-tuning). Our goal in this statement was to propose a possible direction for future research. We have made a minor change in that sentence (line 606) to clarify that this is a possibility from intuition, rather than relying on any

experiments or empirical results from our study.

- Line 578: What is an ‘implicit’ representation? I assume the authors think that what is required is an ‘explicit’ representation? If so, could they tell the reader what they have in mind with these terms?

We have removed these terms from the manuscript and revised the Figure 6 because we found that unpacking them took more space than warranted and the text was more clear without these terms. Thank you!

Specific/minor points:

- Methods: I’m not sure it’s ideal to keep the background elements identical across the two intervals. It adds a change-detection element to the task that is not relevant for contour integration. If change detection contributed to task performance, then I would expect that order of presentation had an effect on performance. Specifically, I would expect that trials, in which the no-contour stimulus was presented on the first interval, would lead to higher performance. If the authors agree with this idea, it might be worth running a quick analysis to see whether this prediction pans out. If it does, I think it’s important to discuss the possibility that performance in human observers in this setup is not exclusively driven by contour detection ability.

We agree that wouldn’t be ideal; luckily, in our behavioral experiment, we made sure the second displays were randomly rotated relative to the first display by 90°, 180°, or 270° before being shown, so that there was no systematic spatial correspondence between elements in the first and second display (and thus, no change detection component). The observers had no knowledge about the relationship between the two displays, and thus had no incentive to look for correspondence between them, since the task was to determine which one had the contour. We have now clarified this in the “*Behavioral Experiment*” section of Methods (line 997-1001).

- Line 209: ‘…the path as compared TO the background…’

FIXED

- Line 249: ‘…dubbed “PinholeNets”,…’ quotation marks are missing after PinholeNets.

FIXED

- I would avoid sentences such as this one in the main text and put it in the figure caption: ‘PinholeNets are illustrated in varying shades of blue (lighter shades correspond to models with smaller receptive 278 fields), while the standard Alexnet model is represented in grey.’

FIXED

- Same as before with sentences like this one: ‘The error bars denote the 95% CI of the mean accuracy for each beta condition bootstrapped across the 78 participants.’

FIXED

- Fig. 5A: I think the beta value for the first contour is incorrect. If it were really beta of zero, the contour should be straight as far as I understand (except for the delta beta value that’s added). I think the depicted contour is probably one with a beta of 15 deg?

- 'i.e.' should be followed by a comma

FIXED

- 2-IFC (Two-Alternative Forced Choice) should be (Two-Interval Forced Choice)

FIXED

- Fig. 5 C and F: you forgot to indicate what the colors mean; why is model performance shown in red in C and in green in F?!? Why are the colors for B and E different.

We have updated the Figure 5 caption to explicitly clarify this distinction. The model depicted in red represents the broadly-tuned model (0-75 degrees), while the green represents the narrowly-tuned model (20 degrees only).

- Red and green are not ideal choices due to the relatively high prevalence of red-green color blindness.

True, in this case no comparisons need to be made that depend on that color pairing so we did not change it for now, but we'll use other colors in the future!

- Line 548: full stop instead of comma

FIXED

- Fig. 2: Mention that contour patches are highlighted for illustration.

FIXED

- Fig. 2A: Why is pre-trained model performance low for deep layers?

One possible explanation for the lower performance of deeper layers in the pre-trained model is that these layers have been shown to encode less shape-based information compared to earlier layers (Hermann et al., 2020). In ImageNet pre-trained networks, deeper layers often prioritize texture-based representations over spatial and shape-related cues. As a result, these layers may be less sensitive to the local alignment signals necessary for contour integration.

- I don't find Fig. 6 helpful at all. In my view, it contains little informative value.

The purpose of this figure is to illustrate two conceptual mechanisms of contour integration: (A) traditional lateral mechanisms that amplify responses to local collinear elements, and (B) the feedforward mechanism proposed in our model, which integrates local features into a complete representation of the extended contour through a hierarchical progression of receptive fields. This figure is meant to complement the discussion by visually distinguishing these approaches. We hope this clarifies the intended role of Figure 6 in the manuscript.

- Reference 59 doesn't have any information beyond author names and title

We have updated that reference (now ref. 62; line 1280-1281).

Muller, N., Groen, I. I., & Scholte, H. S. (2023). Pre-Training on High-Quality Natural Image Data Reduces DCNN Texture Bias. Cognitive Computational Neuroscience Conference.

https://2023.ccneuro.org/proceedings/0000371.pdf

- Line 706: what are ‘current operations’?

Sorry, that was a typo. We meant recurrent operations. FIXED it. Thanks!
