# Supplementary material for: A feedforward mechanism for human-like contour integration
Source: PLoS Comput Biol. 2025 Aug 18;21(8):e1013391. doi: 10.1371/journal.pcbi.1013391 (PMC12370188; doi:10.1371/journal.pcbi.1013391)
Supplement: Attachment — Submitted filename: rebuttal_letter_response_to_reviewers_auresp_2.pdf [file pcbi.1013391.s017.pdf]

HARVARD UNIVERSITY
DEPARTMENT OF PSYCHOLOGY

WILLIAM JAMES HALL
33 KIRKLAND STREET

Fenil R. Doshi
fenil_doshi@fas.harvard.edu

July 25, 2025

Dear Editor,

Thank you for reviewing our manuscript, "***A feedforward mechanism for human-like contour integration***" (PCOMPBIOL-D-24-01619). We have responded to both reviewers' comments and have included our revised manuscript.

In response to Reviewer #1: We conducted their suggested experiment, finding models are in fact using something like good continuation and not a simpler local heuristic. This analysis and its results are presented in a new Supplementary Figure and referenced in the Results section. We believe this new result strengthens our interpretation of the original results and addresses an important alternative hypothesis.

In Response to Reviewer #2: We conducted a new set of experiments on the task of visual uncrowding to directly address the reviewers' concerns about the generality of our findings and the architectural limits of feedforward networks. We now demonstrate that feedforward networks are in fact capable of producing an emergent signature of uncrowding. This key finding is now presented in a new Results subsection, a new Figure, a new Methods subsection, and a new Supplementary Figure detailing the run-to-run variability of this emergent effect. We have incorporated these results throughout the manuscript, including the abstract, author summary, introduction, results, methods, and general discussion.

Please find our point-by-point response to the reviewer's comments, with changes to the manuscript marked in blue. Thank you for your consideration. Please let us know if there is any further information you need.

Sincerely,
Fenil R. Doshi, Talia Konkle and George A. Alvarez

Contact Information:

Fenil R. Doshi	fenil_doshi@fas.harvard.edu	+1 (224) 499-4442
Talia Konkle	talia_konkle@harvard.edu	
George A. Alvarez	alvarez@wjh.harvard.edu	

POINT-BY-POINT RESPONSE TO REVIEWERS
*** Reviewer comments in black and response in red**

Reviewer #1:

I thank the authors for addressing my and the other reviewer's comments on their last submission. Overall, I think the article is improved and close to ready for publication. I do still have a few lingering questions that I would like to see addressed before the article is accepted but I will happily defer to the editor if they feel these questions could be addressed in future research.

Thank you so much! We appreciate the feedback and are glad to know that it has improved the state of the paper.

My biggest question is whether finetuned neural networks could achieve good performance on this task without actually perceiving contours. One possibility that I am not sure is yet ruled out is that networks signal the presence of a contour when some combination of proximity and similar orientation are present. I have tried to illustrate this in the image below. In both sequences of four elements, the orientations are identical and the inter-element distances are (at least approximately) the same. However, humans should get a much stronger perception of contour for the sequence on the left than the right. It's very possible that neural networks do too, but if they don't, that would suggest that they have learned some kind of rule for contour detection based on proximity and orientation similarity rather than good continuation. The authors' discussion of correlated human and network responses to individual trials within curvature conditions is interesting, but without further analysis it's difficult to say why those things correlate.

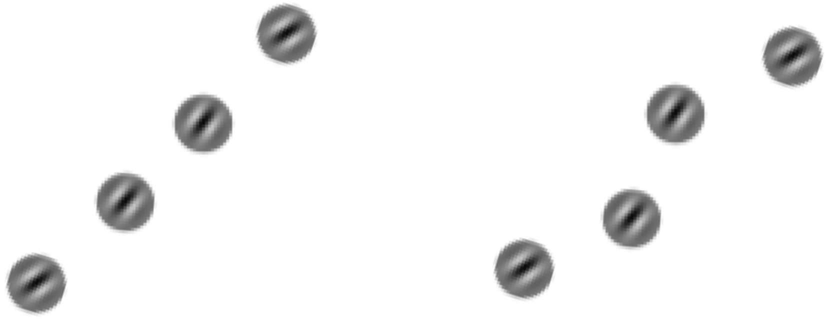

In the revised manuscript we now include this new experiment to address the alternative hypothesis that the models are succeeding through a local strategy based on proximity and orientation similarity, rather than "good continuation" through long-range alignment. To test this, we created a new set of stimuli as suggested by the reviewer, by taking our original contour paths and systematically displacing each contour element perpendicularly from the path by an offset, "alignment jitter" (0-8 pixels). This manipulation degrades the "good continuation" of the path while maintaining local proximity and orientation similarity between the contour elements. We then tested our best performing models on these new jittered stimuli. We find that the model performance systematically reduces as the alignment jitter increases (see results figure below from the broadly-tuned and the most human-like narrowly-tuned model fine-tuned at 20 degree curvature). Critically, at the 8px offset, where

local alignment is completely disrupted (the Gabor elements are ~16px) but local pairwise proximity and similarity is preserved, performance drops to near chance levels. This new finding provides direct evidence that the model is indeed sensitive to the local alignment of the contour elements (good continuation), not just a simple combination of local proximity and orientation similarity. These results are reported in the manuscript in the Results section (*Fine-tuned networks detect contours via integration of locally aligned elements subsection;* line 300-313) and included in a new Supplementary Figure 3.

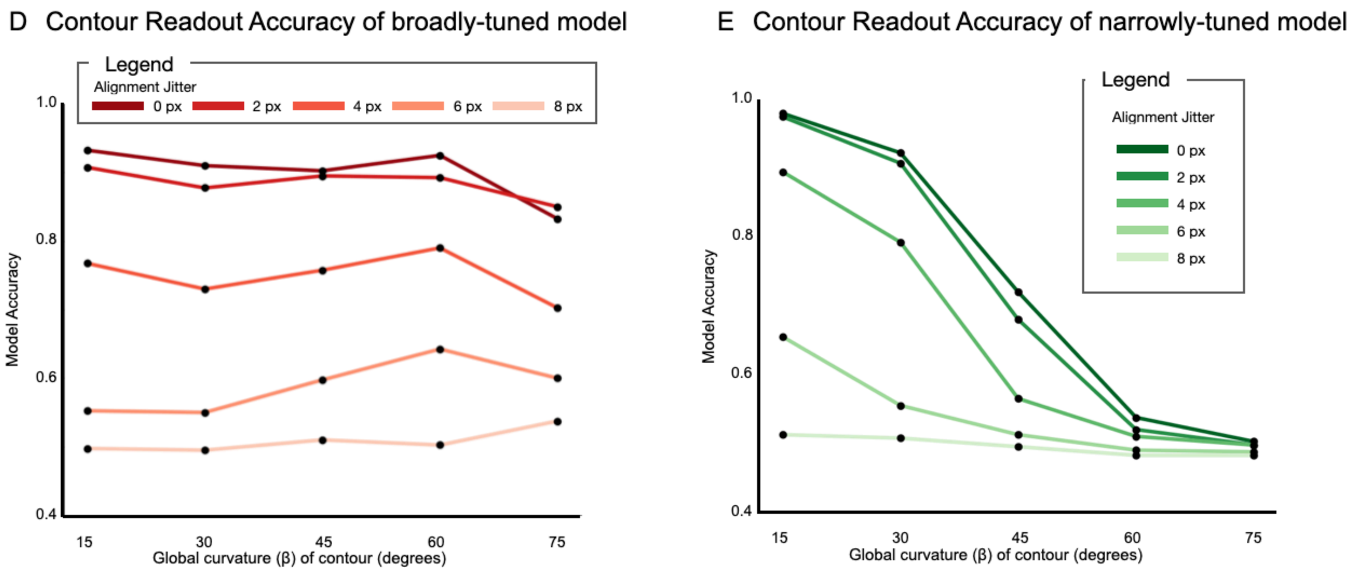

My other comment is simply that the pinhole networks' near-equal accuracy when compared with standard AlexNet is an interesting result on its own that adds to other reports that neural networks are deeply biased to local cues. While it is not the main point of this paper, I'd recommend the authors highlighting this a little more in their discussion.

We have now highlighted this point in the Discussion section (in *Towards Emergent Human-like Behavior subsection* in line 844-852). Thanks!

Reviewer #2:

I have read the response by the Authors and the revised manuscript. I remain disappointed: the authors have done nothing of substance to address my concerns, except including a brief paragraph that essentially says: "There is this far more interesting and recent finding in the perceptual literature (uncrowding) that our model cannot explain, but we don't claim that we can explain it, so we leave it for others to figure out". That's fine, but that's not the standard I expect for PLoS CB (which doesn't mean that this would not be ok in some other journal).

Given your feedback, we have now conducted a new series of experiments on visual uncrowding, to directly test the claims about the architectural limits of feedforward networks. In fact, we actually show that under the right conditions we can unlock the capacity to show emergent uncrowding even in a purely feedforward architecture! These results are presented in a new results section (*Assessing the generality of a feedforward architecture's capacity in other grouping phenomenon;* line 602-670), a new Figure 6, a new methods section (*Assessing the feedforward architecture on visual uncrowding*; line 1237-1261), a new Supplementary Figure 13 , updated the introduction section (line 118-122; line 160-171), updated the discussion section (line 688-691; 755-760; line 783-786; line 888-890), updated the author summary (line 67-68) and the abstract (line 45-48; line 51-54).

Specifically, we directly explored the uncrowding phenomenon from Doerig et al. Critically, we followed a similar analysis plan as we did with assessing contour detection capabilities of the model, which assess not only a frozen backbone but also a fine-tuning protocol. First, just as done in Doerig et. al, given a model trained on Imagenet, we kept the weights *frozen* and trained our read-out layer only using displays where the vernier target and shape flankers were spatially far apart, with no overlap. Then, we probed how well this frozen model could perform the vernier task, now under conditions of crowding with increasing numbers of flankers. Replicating Doerig et al, we saw no evidence of uncrowding.

However, we next followed the same procedure, but allowed *fine-tuning* of the weights while training the read-out module. That is, we kept the training stimuli the same, with no crowded displays—the fine-tuning just allows the model to adjust its purely feed-forward weights to more effectively represent and distinguish these simple stimulus displays. And, the key finding is that we now see evidence of emergent uncrowding! The vernier task is difficult when surrounded by only one flanker, but gets progressively easier with increasing flankers.

This is similar to what we observed with our tests of contour detection and inferences about the capacities of feed-forward computations. Specifically, we found that the model trained only on natural image statistics showed only modest contour detection ability. But, it turns out, this weak capacity is *not* attributable to limits of purely feedforward architectures (as we thought) — when we fine-tuned the model's weights to adjust how it encodes the contour stimuli (which are fairly out of distribution compared to natural images), the model succeeds

at contour detection. There was not an architectural limit there. Apparently the same is true for uncrowding: a model trained only on natural images and frozen does not show uncrowding signatures, but allowing an adjustment to the feed-forward weights does! Therefore, the problem is not architectural (like missing lateral, or feedback connections), but more about challenges of representing out-of-distribution stimuli.

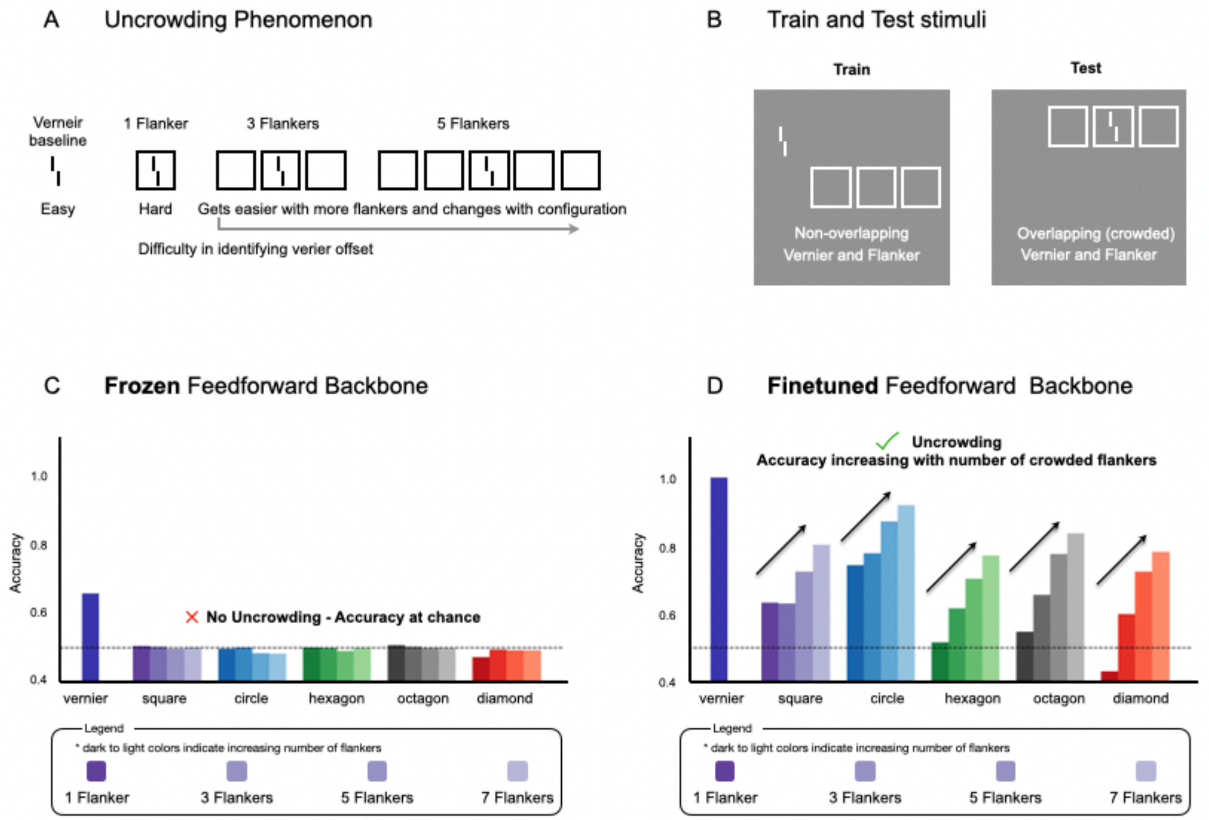

In their reply, the authors imply that I was confused about the fact that their model was never trained on human data. Of course I understood this aspect of their submission, and honestly I don't see how that can be a point of surprise: it is what people have been doing for decades, it is the standard approach of setting up an over-parameterized architecture and finding out which parameterization produces metrics that align with human trends. But that wasn't my point. My point was that the phenomenon at hand is not one which, personally, I would have picked to exercise the power of ffCNNs in 2025. In 2010, maybe. But not in 2025. The authors state: "Specifically, we thought that Alexnets would be architecturally incapable of detecting these contours, much like the claim you highlighted in Doerig's work that "their results provide evidence that ffCNNs cannot produce human-like global shape computations for principled architectural reasons."" This is a misinterpretation of what Doerig et al meant (and I am not one of those authors, but I understand their work). The "human-like global shape computations" those authors were referring to are those involving release from crowding under complex object-based grouping

rules, which extend far beyond contour completion in terms of conceptual significance for contextual vision. If the authors of the paper under review thought that "Alexnets would be architecturally incapable of detecting these contours", in my mind this statement reflects a limitation of their knowledge about this specific area of research in human and machine vision: it would have never occurred to me to even try that, so little is the interest I ascribe to this notion in 2025. In their rebuttal, the authors seem to position snake stimuli alongside uncrowding configurations, as if in both cases they were probing ffCNNs effectively. But this is not at all the case, as I pointed out in my earlier review: it is a straw-man argument. If snake stimuli were on a par with uncrowding, then the authors of this paper would be proving Doerig et al wrong. But they are not, and provide no evidence for that (see para below). And again, I emphasize that I am neither Doerig nor one of his collaborators. I am just a reader who finds the results of Doerig et al interesting, and those in the current submission uninteresting.

We hope you find the addition of these new results more interesting.

What I find more disappointing is that the authors did not make any attempt to explore uncrowding using variations of their approach, if for no other reason, at least to confirm the results/conclusions obtained/reached by Doerig et al (which is always helpful). Instead, they left it as an exercise for others to complete, except the exercise left for others is the very meat of what remains mysterious about contextual vision in 2025.

We hope these new results are as surprising and interesting to the reviewer as they are to us, we think the addition of the uncrowding results substantially increases the breadth of relevance and impact of our work.

I do not expect that we can reach a productive point of discussion here, for the following reason: the material under consideration is perfectly clear to everyone involved, so there is no discussion to be had there. The discussion is not even over whether this material is "a worthy and quality scientific contribution" in the words of the authors: yes there is worth here, not in abundance in my opinion (it is one of dozens of papers we now read every year about network alignment with human behavior), but I am not saying there is none. The discussion is over whether this material makes a significant contribution to our understanding of vision for contemporary readers. As far as this contemporary reader is concerned, this is not an interesting PLoS CB paper---in other words, it has not taught me anything I didn't already know and/or expect based on the work that has already been carried out before (e.g. by Doerig et al). However, if other readers think differently, I will be happy to go with that at this stage: we obviously all have different standards for what is suitable in a journal like PLoS CB, and I am not invested in setting a specific threshold for this journal. My only objective was to encourage the authors to do better than they had done, and challenge their approach with truly interesting problems for vision in 2025, rather than focusing on easy problems. They did not take the challenge, and I think this is disappointing, but I am sure somebody else will go the extra mile in the future.

We heard you and took on the challenge, as well as we could. (Nothing like the review process to push science forward!). While there are likely further cases or arguments to be made or analyses to do, we hope that some of these could be for future work, and that the case we

make here about the (surprising) long-range competencies of feed-forward convolutional architectures, is sufficiently supported for publication in this venue.
