# Supplementary material for: A feedforward mechanism for human-like contour integration
Source: PLoS Comput Biol. 2025 Aug 18;21(8):e1013391. doi: 10.1371/journal.pcbi.1013391 (PMC12370188; doi:10.1371/journal.pcbi.1013391)
Supplement: Attachment — Submitted filename: Review 2.docx [file pcbi.1013391.s016.docx]

I thank the authors for addressing my and the other reviewer’s comments on their last submission. Overall, I think the article is improved and close to ready for publication. I do still have a few lingering questions that I would like to see addressed before the article is accepted but I will happily defer to the editor if they feel these questions could be addressed in future research.

My biggest question is whether finetuned neural networks could achieve good performance on this task without actually perceiving contours. One possibility that I am not sure is yet ruled out is that networks signal the presence of a contour when some combination of proximity and similar orientation are present. I have tried to illustrate this in the image below. In both sequences of four elements, the orientations are identical and the inter-element distances are (at least approximately) the same. However, humans should get a much stronger perception of contour for the sequence on the left than the right. It’s very possible that neural networks do too, but if they don’t, that would suggest that they have learned some kind of rule for contour detection based on proximity and orientation similarity rather than good continuation. The authors’ discussion of correlated human and network responses to individual trials within curvature conditions is interesting, but without further analysis it’s difficult to say why those things correlate.

My other comment is simply that the pinhole networks’ near-equal accuracy when compared with standard AlexNet is an interesting result on its own that adds to other reports that neural networks are deeply biased to local cues. While it is not the main point of this paper, I’d recommend the authors highlighting this a little more in their discussion.
